# Acute impact of afamelanotide on UVR erythemal and melanogenic responses in healthy humans in vivo: an exploratory study

**DOI:** 10.1007/s43630-026-00986-x

**Published:** 2026-09-01

**Authors:** Liezel L. Griffin, Orsolya Kiss, Poonam Halai, Abigail K. Langton, Lesley E. Rhodes, Mark D. Farrar

**Affiliations:** 1 Centre for Dermatology Research, Division of Musculoskeletal and Dermatological Sciences, School of Biological Sciences, Faculty of Biology, Medicine and Health, NIHR Manchester Biomedical Research Centre, The University of Manchester, Manchester, UK; 2 Photobiology Unit, Dermatology Centre, Salford Royal Hospital, Northern Care Alliance NHS Foundation Trust, Manchester Academic Health Science Centre, Manchester, UK

**Keywords:** Afamelanotide, α-melanocyte stimulating hormone, Ultraviolet radiation, Erythema dose response, Melanin density

## Abstract

Afamelanotide, an analogue of α-melanocyte stimulating hormone, activates the melanocortin-1 receptor and has a range of protective properties as demonstrated largely in vitro. While afamelanotide is approved to treat a visible light-induced inflammatory dermatosis, erythropoetic protoporphyria, its acute effects in vivo are poorly characterised. We explored short-term effects of afamelanotide on the acute inflammatory response of UVR-induced erythema and on the melanogenic response, in healthy humans. Participants (*n* = 9, 5 M:4 F, 27-43y, phototypes II-III) had melanin density and skin lightness measured at six skin sites using spectrophotometry. A broadband UVB dose-series (7–80 mJ/cm^2^ erythemally-weighted UVR, Philips TL12) was applied to buttock skin. After 24 h, minimal erythema dose (MED) was assessed, spectrophotometric measurements were taken of erythema at each dose site and two unexposed sites, and an unexposed site was biopsied. Afamelanotide was administered (16 mg subcutaneous implant) and after six days the same UVR dose-series applied to contralateral buttock skin, and measurements and biopsy repeated. Melanin was stained in biopsy sections (modified Warthin-Starry method) and quantified by image analysis. The UVR-erythema dose response (area-under-curve) decreased post-afamelanotide (mean 3.5 pre, 2.7 post, *P* = 0.018) with an apparent increase in MED (median 21 mJ/cm^2^ pre, 29.9 post, not sign.). Melanin density (reflectance at 420 and 400 nm) increased (mean overall increase 2.4% to 2.9%, *P* = 0.001), while melanin staining was unaltered. The demonstrated protection against acute UVR-induced erythema shortly after afamelanotide application supports that anti-inflammatory effects observed in vitro may operate in vivo, warranting further investigation in acute UVR-induced inflammatory conditions.

**Graphical abstract:**

**Figure:**
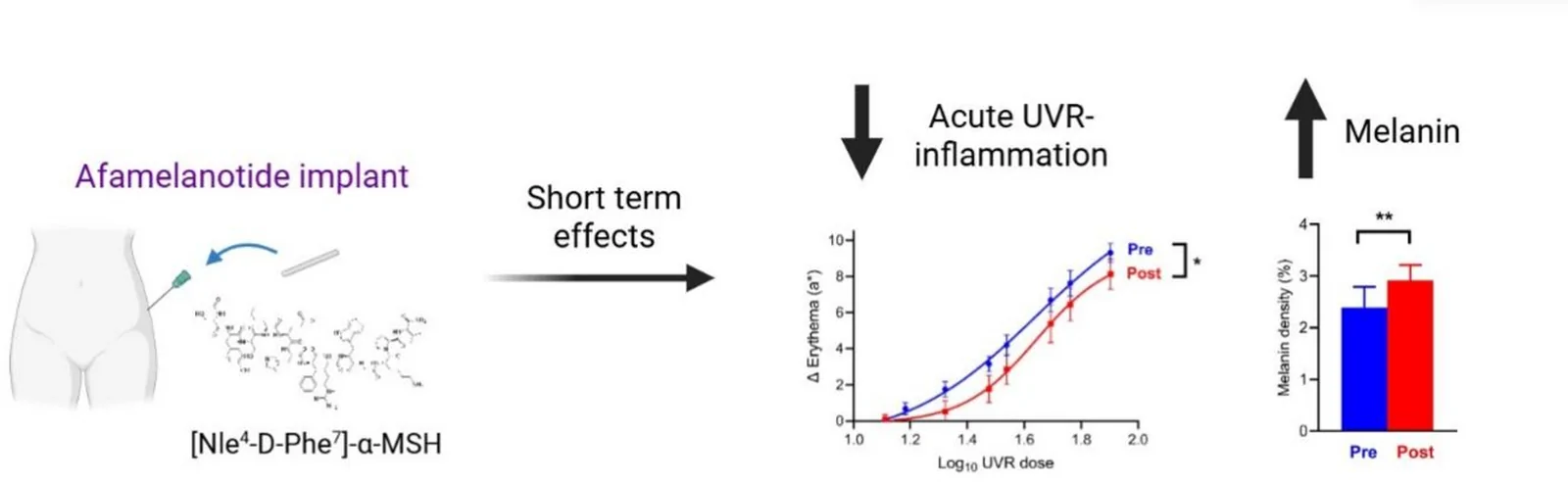

## Introduction

Ultraviolet radiation (UVR) exposure of human skin induces the visible effects of pigmentation (tanning) and erythema, with erythema comprising a component of the acute inflammatory response to UVR. The inflammatory response is mediated by a cascade of signalling pathways including pro-inflammatory cytokines, eicosanoids such as prostaglandin E_2_, nitric oxide, and other reactive oxygen species (ROS), accompanied by a pro-inflammatory leucocytic infiltrate [1–3].

Tanning is triggered by the UVR-induced release of alpha melanocyte stimulating hormone (α-MSH) from keratinocytes, a process involving DNA damage, followed by its binding to the melanocortin 1 receptor (MC1R) on melanocytes. Melanin pigment is produced by melanosomes and transferred to adjacent keratinocytes, providing some protection against further DNA damage by absorbing and scattering UVR [4, 5].

The activation of MC1R by α-MSH is involved in a range of additional protective/reparative activities, including upregulation of DNA damage repair pathways, and antioxidant effects which may protect against DNA damage [6–8] and confer genomic stability in melanocytes in vitro [9]. Anti-inflammatory effects of α-MSH may occur through several mechanisms, as indicated largely by in vitro studies. This includes reduced nuclear translocation of the transcription factor nuclear factor kappa-light-chain-enhancer of activated B cell (NF-κB), leading to down-regulation of interleukin (IL)-1β, IL-6, tumour necrosis factor-α (TNF-α) and chemokine IL-8 [10], upregulation of antioxidant enzymes [11, 12], and prevention of UVR-suppression of nuclear factor erythroid 2-related factor 2 (Nrf2) [13]. Reduced oxidative stress was observed in human melanocytes in vitro [14], while reduced migration of murine neutrophils exposed to α-MSH has been demonstrated [15], and α-MSH inhibited allergic airway inflammation and reduced arthritis in animal models [16].

Synthetic α-MSH analogues have been developed which exhibit increased binding to MC1R, including the analogue [Nle^4^-D-Phe^7^]-α-MSH (afamelanotide). This has been shown to stimulate melanogenesis in humans in vivo, including those of skin types I and II who normally tan poorly on UVR exposure [17]. The UVR-protective effect of afamelanotide-induced melanogenesis was demonstrated in a randomised placebo-controlled study in *n* = 65 healthy white Caucasians [18]. Multiple afamelanotide administrations 0.16 mg/kg/day for three 10-day cycles via subcutaneous injection over 3 months resulted in significantly fewer sunburn cells (apoptotic keratinocytes) following UVR challenge at 90 days compared to baseline.

A controlled release formulation of afamelanotide (Scenesse®, Clinuvel Ltd.) has shown protection in a visible light-induced inflammatory skin disorder, i.e. the photodermatosis erythropoietic protoporphyria (EPP), with regulatory approval gained [5]. A phase 2 open-label pilot study in *n* = 5 EPP patients [19] showed that after 120 days of treatment, patients had a significant increase in melanin density and tolerance to xenon lamp irradiation but interestingly, increased light tolerance did not correlate with increased melanin density [19]. Subsequent international multi-centre phase 3 double blind placebo controlled clinical trials (*n* = 168) confirmed the benefits of afamelanotide in EPP; patients receiving three to five doses, once every 60 days, had a significantly greater duration of pain-free time, fewer phototoxic reactions and improvement in quality of life [5].

Further, a pilot study has suggested benefit in the photodermatosis solar urticaria, which is induced by UVR and visible light [20]. Solar urticaria patients (*n* = 5) administered a single 16 mg subcutaneous dose of afamelanotide showed reduced weal area and increased minimal urticarial dose on monochromator testing, indicating its photoprotective properties in this acute inflammatory disorder [20]. Afamelanotide has further shown promise in a small phase II pilot study (*n* = 3) of inflammatory acne [21] and in *n* = 2 patients with the refractory blistering genodermatosis Hailey Hailey disease [22]. Reduced oxidative stress was observed in vitro in primary keratinocytes of Hailey Hailey disease, following afamelanotide upregulation of Nrf2 [22]. Additionally, Toll-like receptor 2 and 4 mediated inflammatory responses were inhibited in rat microglial cells, leading to reduced translocation of NF-kB and reduced inflammation post-afamelanotide [23].

To date, in vivo studies have examined the effects of afamelanotide after longer-term administration (weeks to months) when the drug is no longer bioavailable. The aims of this exploratory study were to investigate the short-term effects of a single dose of Scenesse® (afamelanotide 16 mg implant) on (i) UVR-induced erythema and (ii) skin melanisation, in healthy humans in vivo.

## Methods

### Study design

**Fig. 1:**
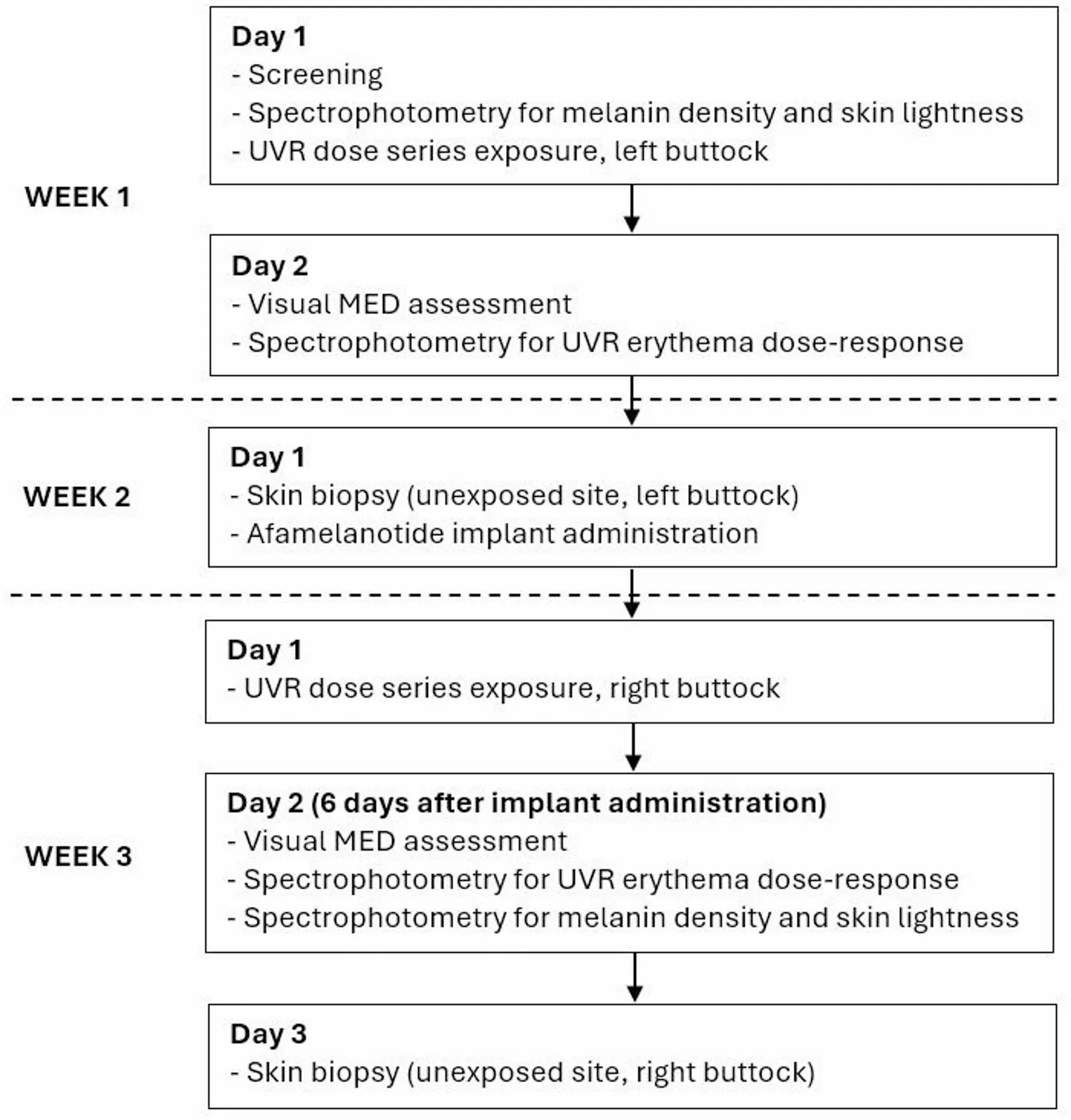
Study protocol flow chart. UVR, ultraviolet radiation; MED, minimal erythema dose

This was an open-label single centre study in *n* = 10 healthy adult volunteers. Participants aged 18–45 years of skin phototype I-III and with a body-mass index between 18 and 30 kg/m^2^ were recruited by open advertisement. Exclusion criteria were previous personal or family history of melanoma, pregnancy/breastfeeding, females of childbearing potential or sexually active males not using adequate contraception, or concurrent use of photosensitising medications. Ethical approval was obtained from the Wales Research Ethics Committee 5 (ref: 20/WA/0315). Participants gave written informed consent and the study adhered to Declaration of Helsinki principles. The study was conducted in the Photobiology Unit, Salford Royal Hospital, Manchester, UK between January and November 2022. Day 6 was chosen for post-afamelanotide evaluation based on the known pharmacokinetics of the drug, where over 90% is released from the implant by day 5 [24]. The study protocol is shown in Fig. 1.

### UVR exposure and erythemal response assessment

A geometric series of erythemally-weighted doses (7–80 mJ/cm^2^) of broadband UVB (Philips TL12, approx. 60% UVB, 40% UVA) was applied to photoprotected upper buttock skin. The minimal erythema dose (MED) was defined as the lowest UVR dose needed to induce just perceptible erythema in human skin at 24 h post-irradiation and was visually assessed by two investigators. Reflectance spectrophotometry (Konica Minolta CM-600D, Tokyo, Japan) was used to measure erythema intensity (CIE a*) in triplicate at each dose site and adjacent unirradiated skin.

### Non-invasive skin pigmentation measurements

Skin lightness (CIE L*) and melanin density [24] were assessed by reflectance spectroscopy (Konica Minolta CM-600D, Tokyo, Japan) at six body sites, including photoexposed and photoprotected sites: forehead, left cheek, right inside upper arm, left medial forearm, right side of abdomen (avoiding implant insertion site) and left buttock. Reflectance measurements were taken in triplicate at each site. Melanin density is calculated from reflectance measurements at 400 and 420 nm; absorption of these wavelengths by haemoglobin is equivalent, whereas melanin absorption is less at 420 nm and carotene does not play a significant role. The difference in absorption between the wavelengths is suggested as a more accurate measure of melanin density, with Dwyer et al. [25] reporting a stronger correlation of melanin density measured histologically with the difference in reflectance at 400 and 420 nm (*r* = -0.68) than with L* (*r* = -0.46).

### Skin biopsy and histological assessment of melanin

Two 6 mm skin biopsies were taken under local anaesthesia from UVR-protected sites, one from the left upper buttock at baseline and one from the right upper buttock six days post afamelanotide administration. Skin Sect. (10 μm) were stained using a modified Warthin-starry method [26]. Stained melanin was quantified in a blinded manner as percentage coverage of the whole epidermal area (excluding *stratum corneum*) using ImageJ software [27]. For each biopsy, three fields of view were captured and measured from each of three biopsy sections.

### Statistical analysis

The sample size was appropriate for an exploratory study. GraphPad Prism (v5.01; GraphPad Software, San Diego, California, USA) was used for all statistical analysis. Statistical significance was accepted at *P* < 0.05. Difference in MED pre and post treatment was analysed using the Wilcoxon signed rank test. The effect of afamelanotide on the UVR-erythema dose-response (assessed as area under curve, AUC), melanin density and L* was examined using a paired T-test or non-parametric Wilcoxon signed rank test as appropriate.

## Results

### Study participants

Ten volunteers were recruited to the study. One withdrew between implant administration and the post-treatment series of UVR exposures due to medical issues unrelated to the study drug or procedures. Baseline characteristics of completing participants are shown in Table 1. Afamelanotide was well tolerated; two participants reported mild headache for 24–48 h after implantation, one reported mild nausea. Three participants experienced mild bruising to biopsy sites and two reported erythema, irritation or weeping.

**Table 1:** Baseline characteristics of completing participants (*n* = 9)

Age (y)^a^	35.3 + 5.1 (27–43)
Sex M: F (n)	5:4
BMI (kg/m^2^)^a^	24.5 + 3.5 (18.5–29.3)
Skin type II: III (n)	5:4
MED (mJ/cm^2^)^b^	21.0 (12.9–29.9)

^a^ Mean + SD (range)

^b^ Median (range)

### UVR erythema responses

**Fig. 2:**
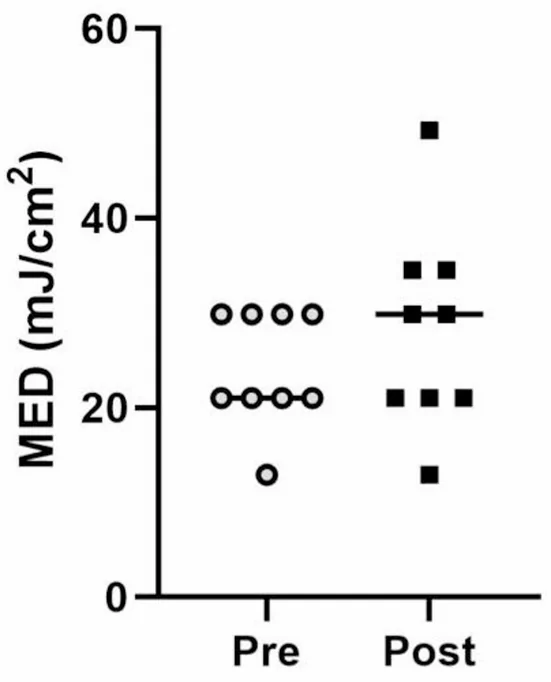
Individual MED (mJ/cm^2^) pre- and 6 days post-afamelanotide implantation (*n* = 9, bar represents the median)

**Fig. 3:**
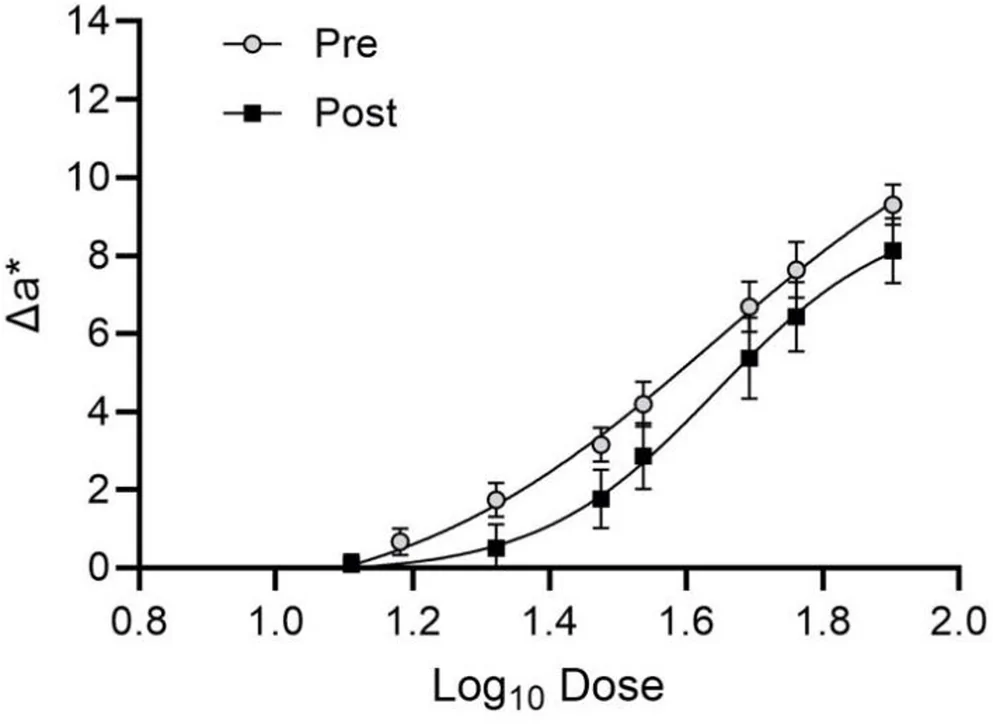
UVR-erythema dose response pre- and 6 days post-afamelanotide administration. Δa*, difference in erythema intensity between UVR-exposed site and adjacent unexposed skin (*n* = 9; data are mean ± SEM)

An increase in the visual erythema threshold was seen post-afamelanotide with median MED 29.9 mJ/cm^2^ (IQR 21.0-34.5) compared to 21.0 mJ/cm^2^ (IQR 21.0-29.9) pre-afamelanotide but this difference was not statistically significant (Fig. 2). However, the overall UVR-erythema dose response, measured as AUC, was significantly reduced post-afamelanotide administration (mean ± SD 2.7 ± 1.5) compared to pre-afamelanotide (3.5 ± 0.9, *P* = 0.018; Fig. 3).

### Skin pigmentation

**Fig. 4:**
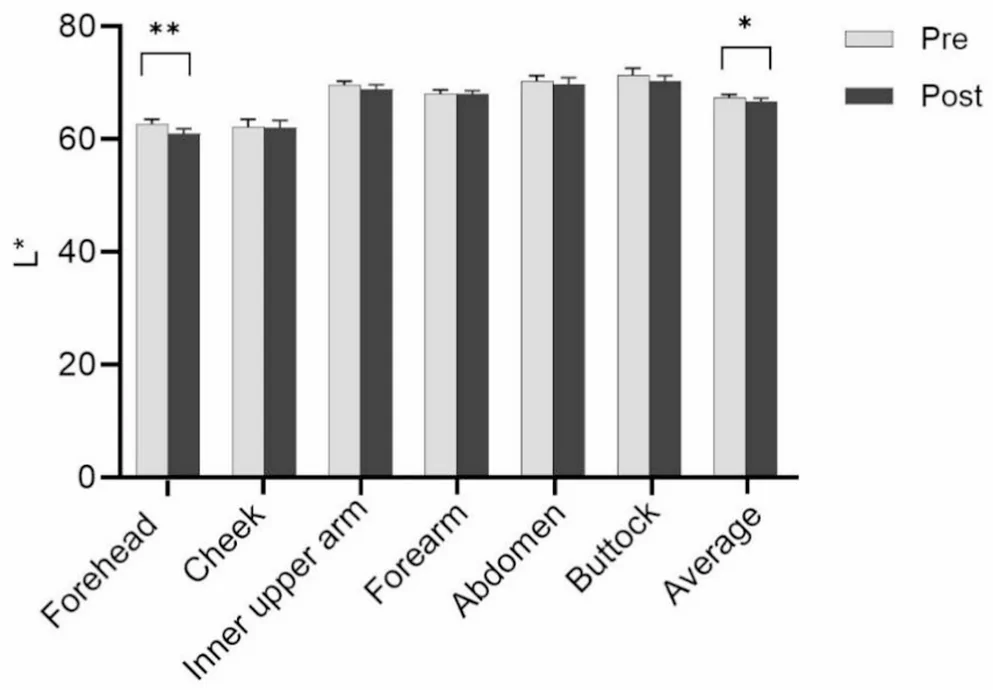
Skin lightness (CIE L*) pre- and 6 days post-afamelanotide administration (*n* = 9; data are mean ± SEM; **P < 0.01, *P < 0.05)

**Fig. 5:**
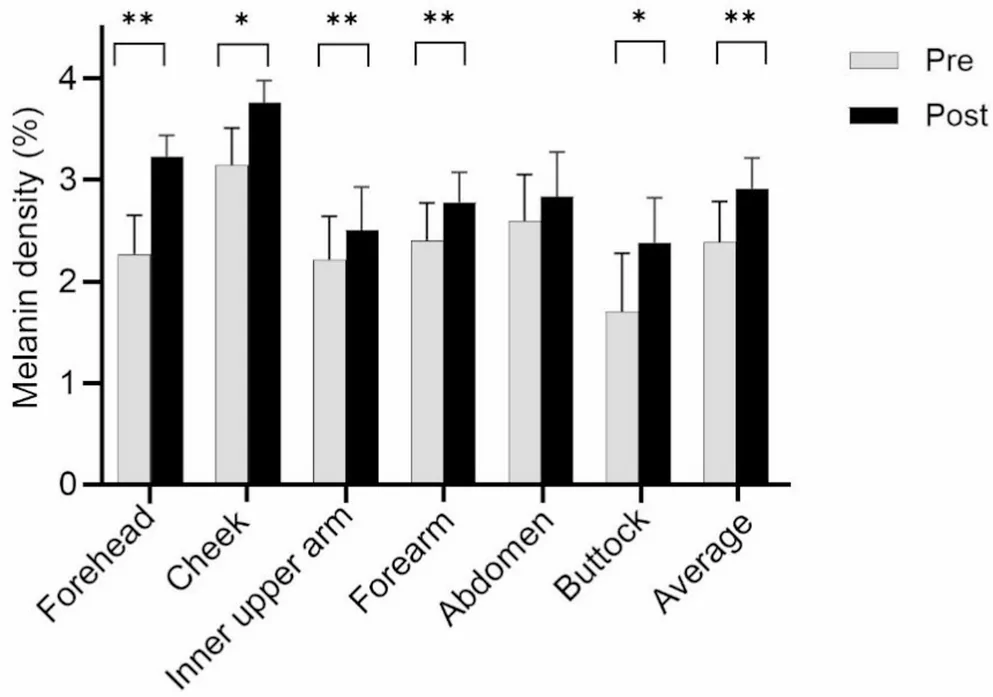
Melanin density (%) pre- and 6 days post-afamelanotide administration (*n* = 9; data are mean ± SEM; **P < 0.01, *P < 0.05)

Compared to skin pre-afamelanotide, skin lightness (CIE L*) post-afamelanotide showed a small but significant decrease (i.e. darkening) at the central forehead site (mean ± SD pre 62.7 ± 2.7 vs. post 61.0 ± 2.6, *P* = 0.0015) and for the average values from all sites combined (pre 67.4 ± 1.7 vs. post 66.7 ± 1.7, *P* = 0.039; Fig. 4). Spectrophotometric melanin density was calculated for each site and compared to pre-afamelanotide, values post-afamelanotide were significantly higher at all sites except the abdomen, and for the average values from all sites combined which increased from pre 2.4% ± 1.2 pre to 2.9% ± 0.9 post (*P* = 0.001; Fig. 5). Quantification of stained melanin in skin biopsy sections as percent epidermal area covered showed no significant change at six days post-afamelanotide (mean 0.7 ± SD 0.42%) compared to pre-afamelanotide (mean 0.9 ± SD 0.7%).

## Discussion

The short-term effects of afamelanotide post-application have not previously been described in human skin. This study is novel in evaluating the short-term impact of a single dose of afamelanotide on the UVR-erythema (sunburn) and pigmentary responses in healthy humans in vivo. We found that six days post-administration of afamelanotide there was a significant reduction in the overall UVR-erythema dose response (AUC) compared to baseline. The apparent small increase in median MED post-administration was in keeping with this finding. The step increments in the UVR doses means that modest changes may not be detected by the visual MED assessment, which is also a subjective method [28], and smaller effects are better demonstrated by the UVR-erythema dose-response curve, which here indicated a significant reduction in this acute skin inflammatory response.

To assess any short-term effects of afamelanotide on skin pigmentation, which indicate MC1R stimulation and could contribute to UVR-erythema protection, we used spectroscopic methods to measure skin lightness (CIE, L*) and calculated melanin density according to Dwyer et al. [25]. We found a small but significant increase in melanin density at all skin sites measured except the abdomen, and for the average of all sites combined. Measurement of L* revealed a significant decrease in skin lightness when all sites were combined, supporting the finding for melanin density, but for individual sites only the central forehead showed a significant decrease. This may be attributable to L* encompassing reflectance across the visible light range (400–700 nm) where in addition to melanin, other chromophores including carotene and haemoglobin contribute [25].

In contrast, our quantification of melanin in buttock skin by modified Warthin-Starry staining with image analysis did not show any measurable increase post-afamelanotide. This method is reportedly more sensitive and specific for melanin in human skin than the Fontana-Masson and Von-Kossa stains [26]. More robust melanin quantification techniques, using a combination of histological stains with high performance liquid chromatography [29], could improve our understanding of the short-term melanisation effect of synthetic α-MSH analogues [30]. The disparity in melanin detection can be explained, as non-invasive spectroscopy measurements encompass a larger surface area, thereby detecting smaller changes in melanisation in comparison with staining of skin sections a few microns thick. This illustrates the utility of incorporating this non-invasive measure.

Barnetson et al. [18] reported significantly decreased L* and increased melanin density in vivo at all body sites at 90 days. However, their participants received 0.16 mg/kg of afamelanotide via subcutaneous injection daily for 30 days in total at different time points across the study period, compared to a single 16 mg subcutaneous implant in ours. Most afamelanotide is released from the implant within 48 h of administration and 90% by day five, but it can be detected in plasma for up to ten days [24]. Afamelanotide is therefore bioavailable for MC1R binding during this period and subsequent effects are largely due to the initial receptor binding in the first five days post-implantation. The route of administration and repeated dosing reported in previous studies [17, 18] likely prolonged the melanogenic effects of afamelanotide, accounting for the substantial change in melanin density and skin lightness.

While it is conceivable that the small increases in melanin density observed in our study following afamelanotide administration could provide some increased protection against UVR-erythema, other, i.e. non-melanogenic properties of afamelanotide may be contributing to the reduction in this skin inflammatory response. Short-term in vivo anti-inflammatory effects induced by α-MSH binding to MC1R may involve downregulation of pro-inflammatory cytokines via suppression of NF-κB translocation [10, 11], reduced chemotaxis of neutrophils from dermal blood vessels through suppression of IL-8 [2] - similar to the sebocyte response to afamelanotide in inflammatory acne [11, 23], decreased vasodilation of dermal blood vessels due to downregulation of prostaglandin E_2_ in keratinocytes, and counteraction of ROS by increased Nrf2 transcription factor activity [1–3, 31].

A limitation of this study was the small number of participants (*n* = 9), but a larger number would be challenging to recruit for an intensive study including skin biopsies. In vivo studies published to date have assessed the impact of afamelanotide weeks to months after administration [17, 18, 29]. This is the first in vivo investigative study examining the short-term effects of afamelanotide on the UVR-erythemal response and melanogenesis. Further to this exploratory study, in vivo research including UVR-exposed skin biopsies may evaluate the short-term impact of afamelanotide on UVR-induced tissue including DNA damage markers.

This study demonstrated protection against acute UVR-induced erythema shortly after afamelanotide application, with modest impact on melanin density, supporting that anti-inflammatory effects of α-MSH analogues observed in vitro may be operating *in vivo.* This warrants further investigation in humans, including in acute UVR-induced inflammatory conditions.

## Data Availability

The data that support the findings of this study are available from the corresponding author upon reasonable request.
